# The Speed of Increasing milk Feeds: a randomised controlled trial

**DOI:** 10.1186/s12887-017-0794-z

**Published:** 2017-01-28

**Authors:** Jane Abbott, Janet Berrington, Ursula Bowler, Elaine Boyle, Jon Dorling, Nicholas Embleton, Edmund Juszczak, Alison Leaf, Louise Linsell, Samantha Johnson, Kenny McCormick, William McGuire, Tracy Roberts, Ben Stenson, The Sift Investigators Group

**Affiliations:** 1 BLISS, London, UK; 2 Newcastle Neonatal Service, Royal Victoria Infirmary, Newcastle upon Tyne, UK; 3 Clinical Trials Unit, National Perinatal Epidemiology Unit, Oxford University, Oxford, UK; 4 Department of Health Sciences, University of Leicester, Leicester, UK; 5 Division of Child Health, Obstetrics and Gynaecology, School of Medicine, University of Nottingham, Nottingham, UK; 6 Child Health, Southampton, UK; 7 John Radcliffe Hospital, Oxford, UK; 8 Centre for Reviews and Dissemination, University of York, York, UK; 9 Health and Population Sciences, University of Birmingham, Birmingham, UK; 10 Simpson Centre for Reproductive Health, Royal Infirmary of Edinburgh, Edinburgh, UK

**Keywords:** Preterm infants, Milk feeds, Milk volume, Prematurity, NEC, Sepsis, Parenteral nutrition, Neurodevelopment, RCT

## Abstract

**Background:**

In the UK, 1–2% of infants are born very preterm (<32 weeks of gestation) or have very low birth weight (<1500 g). Very preterm infants are initially unable to be fed nutritional volumes of milk and therefore require intravenous nutrition. Milk feeding strategies influence several long and short term health outcomes including growth, survival, infection (associated with intravenous nutrition) and necrotising enterocolitis (NEC); with both infection and NEC being key predictive factors of long term disability. Currently there is no consistent strategy for feeding preterm infants across the UK. The SIFT trial will test two speeds of increasing milk feeds with the primary aim of determining effects on survival without moderate or severe neurodevelopmental disability at 24 months of age, corrected for prematurity. The trial will also examine many secondary outcomes including infection, NEC, time taken to reach full feeds and growth.

**Methods/design:**

Two thousand eight hundred very preterm or very low birth weight infants will be recruited from approximately 30 hospitals across the UK to a randomised controlled trial. Infants with severe congenital anomaly or no realistic chance of survival will be excluded. Infants will be randomly allocated to either a faster (30 ml/kg/day) or slower (18 ml/kg/day) rate of increase in milk feeds. Data will be collected during the neonatal hospital stay on weight, infection rates, episodes of NEC, length of stay and time to reach full milk feeds. Long term health outcomes comprising vision, hearing, motor and cognitive impairment will be assessed at 24 months of age (corrected for prematurity) using a parent report questionnaire.

**Discussion:**

Extensive searches have found no active or proposed studies investigating the rate of increasing milk feeds. The results of this trial will have importance for optimising incremental milk feeding for very preterm and/or very low birth weight infants. No additional resources will be required to implement an optimal feeding strategy, and therefore if successful, the trial results could rapidly be adopted across the NHS at low cost.

**Trial registration:**

ISRCTN Registry; ISRCTN76463425 on 5 March, 2013.

## Background

### Outcomes affected by feeding strategies

In the UK, 1–2% of newborn infants are very preterm or have very low birth weight. Preterm birth is the major risk factor for infant mortality, with 73% of neonatal deaths in the UK occurring in infants born before 37 completed weeks of gestation [1]. As survival, especially of very preterm infants, has increased in recent years [2], the high prevalence of morbidity associated with preterm birth means that the assessment of long-term outcomes has become increasingly important [3].

Short and long-term outcomes for preterm infants are affected by strategies that reduce infection rates, lower NEC rates, promote adequate growth, and maintain access to tertiary level facilities. Optimising feeding strategies affects all of these outcomes. Benefits are therefore likely to arise both from the individual and combined effects of identifying the optimum feeding strategy, as the rates of such complications in very preterm infants are high. NEC severe enough to cause death or require surgery affects approximately 7.5% of infants born before 29 weeks gestation, and is the cause of death in 11% of the deaths of infants born before 32 weeks [4]. Late-onset infection affects around 25% of very preterm infants and is responsible for 10% of deaths in the same population. Long-term data following late onset infection or NEC suggest these conditions almost double the risk of poor neurodevelopmental outcome [5].

### Nutritional support of preterm infants and speed of increasing milk feeds

Every year in the UK around 8000 infants are born so preterm that they cannot initially be fed milk and therefore require intravenous nutrition. Milk feeding is gradually increased as the immature gut begins to tolerate milk and intravenous nutrition is correspondingly reduced, but there are few data determining how quickly this is best achieved [6].

One of the most serious complications of intravenous feeding is late-onset sepsis, which occurs in 27% of infants born weighing less than 1500 g at birth or under 29 weeks’ gestation [6]. Late-onset sepsis is known to cause poor long-term cognitive outcomes, liver damage and also sudden death from cardiac problems resulting from misplaced catheters [7–9]. One of the most common late-onset infections is ‘catheter-related bloodstream infection’; the risk of bloodstream infection being directly related to the time the catheter is indwelling in the bloodstream [10–12].

The more rapid advancement of enteral feeds described in this study will, in principle, reduce exposure to intravenous nutrition by causing infants to reach full milk feeds (tolerating 150 ml/kg/day for 3 consecutive days) about 4 days earlier than the slower advancement. Reducing exposure by this amount could reduce the number of infections by between 5 and 15 cases per 250 infants, which is an absolute risk reduction of 4%. This is possibly an underestimate of the reduction as infection risk increases with the length of time a catheter is in place [13, 14].

However, faster increases in milk feed volumes may increase the likelihood of NEC which, as well as being potentially fatal, may provoke intolerance of feeds or gut dysfunction that could result in longer times to achieve full feeds rather than shorter. Survivors of NEC also have significantly worse long-term outcomes across multiple developmental domains than those unaffected [5, 15].

Therefore, while emerging data suggest better health outcomes may be achieved with faster feeding increments, there are possible disadvantages and a randomised controlled trial is required to support a change in clinical practice [6].

### Trial objectives

The primary objective of the trial is to compare the effects of two speeds of increasing milk feeds on survival without moderate or severe disability at 24 months of age (corrected for prematurity). The secondary objectives are to assess the impact of the two speeds of increasing milk feeds on the incidence of sepsis and NEC, and other outcomes collected before hospital discharge.

## Methods/design

The trial is a multi-centre randomised controlled group trial (RCT) to assess whether the speed of increasing milk feed volumes (faster increase [30 ml/kg/day] versus slower increase [18 ml/kg/day]) in very preterm (<32 weeks) or very low birth weight infants (<1500 g) infants has any effect on survival without moderate or severe disability at 24 months corrected age.

Two thousand eight hundred infants from approximately 30 neonatal units will be recruited within the UK and Ireland over 3 years.

### Inclusion criteria

- Gestational age at birth is <32 completed weeks, or birth weight <1500 g
- Receiving ≤30 ml/kg/day of milk at randomisation
- Written informed parental consent is obtained

### Exclusion criteria

- Severe congenital anomaly
- In the opinion of the treating clinician, have no realistic chance of survival
- Unlikely to be traceable for follow-up at 24 months of age (for example, infants of non-UK residents)

### Primary outcome

The primary outcome will be the proportion of infants surviving without moderate or severe neurodevelopmental disability at 24 months of age corrected for prematurity. This composite outcome will be determined by confirming that the child is alive or dead using records held and maintained by The Health and Social Care Information Centre and other central UK NHS bodies. For live infants, a parent report questionnaire will be used to assess sensory and gross motor impairment and standardised measures to assess cognitive function in order to identify children with:

- Moderate/severe visual impairment (reduced vision uncorrected with aids; or blind in one eye with good vision in the contralateral eye; or blind/perceives light only)
- Moderate/severe hearing impairment (hearing loss corrected with aids; or some hearing loss but not corrected by aids; or deaf)
- Moderate/severe gross motor impairment (unable to walk or sit independently)
- Moderate/severe cognitive impairment assessed using the Parent Report of Children’s Abilities–Revised (PARCA–R), a parent report measure of non-verbal cognitive and language development. Total PARCA–R scores <44 will be used to identify children with moderate/severe cognitive impairment [16]. This questionnaire has been shown to have at least 80% sensitivity and 80% specificity for identifying children with scores < −2SD on a Gold Standard development test [16, 17]

A child who has any one or more of these impairments will be classified with a moderate/severe disability. Definitions for motor and sensory impairments described above are as defined in the report published by British Association of Perinatal Medicine (BAPM) in 2008 [18].

### Secondary outcomes

Secondary outcomes to be assessed when infants are discharged home for the first time are:

- Survival to discharge home.
- Microbiologically-confirmed or clinically suspected late-onset invasive infection from trial entry to discharge home.
- NEC (Bell stage 2 or 3) from trial entry to discharge home.
- Time taken to reach full milk feeds (tolerating 150 ml/kg/day for 3 consecutive days).
- Growth (change in z score–weight and head circumference for gestational age) from birth to discharge home.
- Duration of parenteral feeding.
- Length of time in intensive care.
- Length of hospital stay to discharge home.

In addition, the separate components of the composite primary outcome at 24 months of age corrected for prematurity will be analysed individually as secondary outcomes. The diagnosis of cerebral palsy by a doctor or other health professional will also be a secondary outcome assessed at this age.

### Trial procedures

#### Informed consent

Written consent will be sought from parents only after they have been given a full verbal explanation and written description of the trial via a parent information leaflet. Consent will be obtained by means of dated parental signature on a study consent form and the signature of the person who obtained informed consent. Recruitment will be conducted by a health professional with delegated authority. Parents who do not speak English will only be approached if an interpreter is available.

### Remuneration

Parents will not be given any financial or material incentive or compensation for enrolling their babies in this trial.

### Randomisation and allocation

Randomisation will take place at the time the clinician is ready to start increasing the feed volume. This will be performed through a secure website hosted by the NPEU CTU with telephone back-up available 24/7, 365 days a year.

A minimisation algorithm will be used to ensure balance on important prognostic factors: hospital, multiple birth, gestational age ranges, and birth weight <10th centile for gestational age. Multiple births will be given the same allocation.

### Blinding

This is an open trial; blinding of the clinicians, nursing staff, and parents is not possible. A blinded endpoint review committee will examine the relevant data collection forms and clinical notes of infants with possible sepsis and NEC and classify them systematically according to predefined criteria.

### Stopping or modifying the trial intervention

Deviations from the scheduled speed of increase may be made at the discretion of the treating clinician if the infant appears unable to tolerate the allocated speed of milk feed increase.

### Withdrawal

At all stages it will be made clear to the parents that they are free to withdraw their infant from the trial at any time, without the need to provide explanation. If parents choose to withdraw, they will be asked for permission to complete data collection and/or follow-up.

The attending clinician may also withdraw the infant from the allocated treatment if they consider this to be in the best interest of the infant’s health and well-being.

### Data collection before discharge

All outcome data are routinely recorded clinical items that can be obtained from the clinical notes or local microbiological laboratory records. No additional blood or tissue samples are required for this trial. Clinical information will be collected using specially created data collection forms.

### Data collection after discharge

A parent questionnaire will assess neurodevelopmental outcomes and health care costs when the infant is 24 months of age (corrected for prematurity).

### Data collection and processing

Data will be processed using validated data management systems to ensure consistency, viability and quality of data. It will be stored in line with the Data Protection Act 1998.

### Safety reporting

Adverse events are defined as serious if they:

- Result in death
- Are life-threatening
- Require inpatient hospitalisation or prolongation of existing hospitalisation
- Result in persistent or significant disability/incapacity, or
- Are a congenital anomaly/birth defect

The term “life-threatening” refers to an event in which the child was at risk of death at the time of the event; it does not refer to an event which hypothetically might have caused death if it were more severe. SAEs are to be reported from randomisation until discharged home.

Safety reporting will be carried out in accordance with the NPEU Clinical Trials Unit standard operating procedures and clinical regulations.

### Sample size and power

The primary comparison will be the difference in the proportion of infants surviving without moderate or severe disability at 24 months of age corrected for prematurity. Based on previous trials, it is estimated that 80% of the infants will survive to 2 years, and that 11% of these will have a moderate or severe disability [19]. Hence it is estimated the proportion surviving without moderate or severe disability in the control group receiving the 18 ml/kg/day increment will be 71%. With a total sample size of 2500 and allowing for a response rate of 80%, there will be 90% power to detect an absolute difference of 6.3% (from 71.0% in the control group to 77.3%) in this proportion, with a two-sided 5% significance level.

With the same level of significance, a sample size of 2500 infants will have 90% power to detect an absolute risk difference of 5.4% (from 25.0% in control group to 19.6%) in the incidence of sepsis [20] and an absolute risk difference of 3.5% (from 6.0% in control group to 9.5%) in the incidence of NEC (Bell stage 2 or 3) [21–23].

An inflation factor of 1.12 was applied to the sample size to allow for multiple births, expected to have correlated outcomes, receiving the same allocation. This was based on an estimate of 25% for the proportion of multiple births and an intraclass correlation coefficient of 0.9 for the primary outcome at 2 years, based on a similar outcome in a preterm population [24]. The total number of babies that will be recruited will be 2800.

### Statistical analysis

Demographic factors, clinical characteristics and outcomes will be summarised with counts (percentages) for categorical variables, mean (standard deviation) for Normally distributed continuous variables, or median (interquartile or entire range) for other continuous variables. Infants will be analysed according to allocation regardless of the speed of milk feed increase they actually receive.

The two groups will be compared using generalised estimating equations, adjusting for the minimisation factors to account for the correlation between treatment groups. This method of analysis will also account for the correlation in outcomes between twins and siblings born in a subsequent pregnancy during the trial period. For the primary outcome, an adjusted risk ratio with 95% confidence will be calculated using log binomial regression, or log poisson regression with a robust variance estimator if the binomial model fails to converge [25]. Linear regression will be used for Normally distributed outcomes, quantile regression for skewed continuous variables, and Cox regression for time to event outcomes. Ninety-nine percent confidence intervals will be calculated for all secondary outcomes.

The consistency of the effect of advancing milk feeds across specific subgroups of infants will be assessed using the statistical test of interaction. Pre-specified subgroup analyses include: (i) week of gestation at birth, (ii) birth weight (<10th centile for gestational age versus ≥ 10th centile and (iii) type of milk (breast milk only/formula only/mixed). Subgroup analysis will be performed on the primary outcome, and the incidence of sepsis and NEC.

### Economic data collection

Relevant resource use data collection will be undertaken prospectively from centres participating in the trial. Private out of pocket costs to parents will be collected via a questionnaire sent at 24 months of age corrected for prematurity. Unit costs will be obtained from published sources and centres participating in the trial and applied to resource use. Published sources will include Unit Costs of Health and Social Care [26] and NHS Reference Costs.

The main economic analysis will be in the form of a cost-effectiveness analysis from the perspective of the health care provider (National Health Service), based on an intermediate outcome of cost per neonatal sepsis avoided when discharged home and on the outcome of disability-free survival at 24 months of age corrected for prematurity (cost per additional survivor without disability at 24 months of age corrected for prematurity). A secondary analysis will extend the perspective to include private out of pocket costs to families associated with travel and time off work during the period of follow up.

The analyses will adopt an incremental approach in that data collection will concentrate on resource use and outcome differences between trial arms. A bootstrapping approach will be undertaken in order to calculate confidence intervals around the mean costs [27, 28]. Costs and benefits will be discounted as per NICE guidelines at 3.5%.

Results will be presented using cost-effectiveness acceptability curves. The robustness of the results will be explored using deterministic and probabilistic sensitivity analysis.

## Discussion

Preterm infants are at significant risk of poor long-term neurodevelopmental problems with almost 12% having moderate or severe disability [19], with both sepsis and NEC dramatically increasing this risk [29–34]. Achieving full milk feeding sooner is associated with significant cost savings through decreased use of intravenous nutrition, a reduction in time spent in a specialist tertiary neonatal unit, shortened total hospital stay (potentially saving £1000 per day), and reductions in societal costs due to improved long-term outcomes [29–34]. Vacating tertiary level neonatal cots sooner will also improve the family’s experience and the infant’s safety by decreasing the need for transfer to other hospitals for intensive care.

Infection and NEC remain highly predictive factors for neurodevelopmental disability. Any reduction in either problem may therefore be expected to reduce long-term disability in this population.

Overall lifetime financial costs of disability are significant, and so preventing even a few cases and reducing cognitive problems at the population level would reduce the financial burden of long-term care for the NHS and society. No additional resources will be needed to implement the optimal feeding strategy, which, if successful, could be adopted rapidly across the NHS at low cost.

## Abbreviations

HTA: Health Technology Assessment
ISRCTN: International Standard Randomised Controlled Trial Number
NEC: Necrotising enterocolitis
NHS: National Health Service
NIHR: National Institute for Health Research
PARCA–R: Parent Report of Children’s Abilities–Revised
SAE: Serious Adverse Event
SIFT: Speed of Increasing Milk Feeds Trial
